# Supplementary material for: Chemical-genetic profiling reveals limited cross-resistance between antimicrobial peptides with different modes of action
Source: Nat Commun. 2019 Dec 16;10:5731. doi: 10.1038/s41467-019-13618-z (PMC6915728; doi:10.1038/s41467-019-13618-z)
Supplement: Supplementary file 1 — Supplementary Info [file 41467_2019_13618_MOESM1_ESM.docx]

**Supplementary information**

**Chemical-genetic profiling reveals limited cross-resistance between antimicrobial peptides with different modes of action**

Bálint Kintses^1,2,3^*^§^, Pramod K. Jangir^1,4§^, Gergely Fekete^5,1§^, Mónika Számel^1,4^, Orsolya Méhi^1^, Réka Spohn^1^, Lejla Daruka^1,4^, Ana Martins^1^, Ali Hosseinnia^6^, Alla Gagarinova^7^, Sunyoung Kim^6^, Sadhna Phanse^6^, Bálint Csörgő^1,8^, Ádám Györkei^5,1^, Eszter Ari^5,1,9^, Viktória Lázár^1,10^, István Nagy^11^, Mohan Babu^6^, Csaba Pál^1^* & Balázs Papp^5,1^*

^1^Synthetic and Systems Biology Unit, Institute of Biochemistry, Biological Research Centre, 6726 Szeged, Hungary

^2^HCEMM-BRC Translational Microbiology Lab, 6726 Szeged, Hungary

^3^Department of Biochemistry and Molecular Biology, University of Szeged, 6726 Szeged, Közép fasor 52, Hungary

^4^Doctoral School of Biology, Faculty of Science and Informatics, University of Szeged, Szeged, Hungary

^5^HCEMM-BRC Metabolic Systems Biology Lab, 6726 Szeged, Hungary

^6^Department of Biochemistry, University of Regina, Regina, Saskatchewan S4S 0A2, Canada

^7^Department of Biochemistry, University of Saskatchewan, Saskatoon, Saskatchewan S7N 5E5, Canada

^8^Present address: Department of Microbiology and Immunology, University of California,

San Francisco, CA 94143, USA.

^9^Department of Genetics, Eötvös Loránd University, 1117 Budapest, Hungary

^10^Present address: Faculty of Biology, Technion – Israel Institute of Technology, Haifa, Israel

^11^Sequencing Platform, Institute of Biochemistry, Biological Research Centre, 6726 Szeged, Hungary

*Correspondence to cpal@brc.hu, pappb@brc.hu or kintses.balint@brc.hu

^§^These authors contributed equally to this work.

**Supplementary Figures**


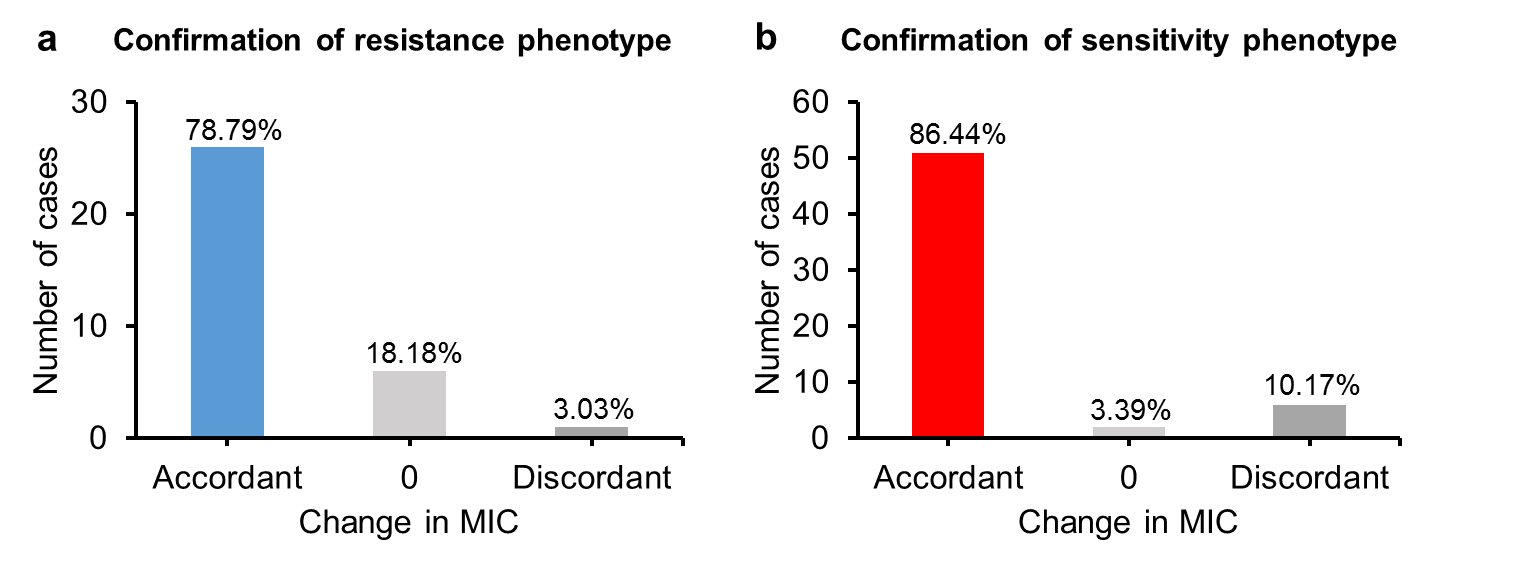


**Supplementary Figure 1. Validation of the chemical-genetic interactions.** We selected 19 overexpression strains and measured the minimum inhibitory concentration (MIC) of the corresponding AMPs for these strains. In total *n* = 92 MIC measurements were performed. **a,** Out of the 33 resistant chemical-genetic interactions, 26 (78.79% true positive) were confirmed by the MIC measurements, while a single case showed an opposite effect. **b,** In the case of sensitive chemical-genetic interactions, out of 59 cases, 51 (86.44% true positive) showed the expected MIC change. Although, an MIC change (difference in the minimum inhibitory concentrations) is not necessary expected upon a chemical-genetic interaction (i.e. growth rate change) at a sub-inhibitory dosage, we could still confirm 83% of the observed chemical-genetic interactions with this complementary approach. Source data are provided as a Source Data file.


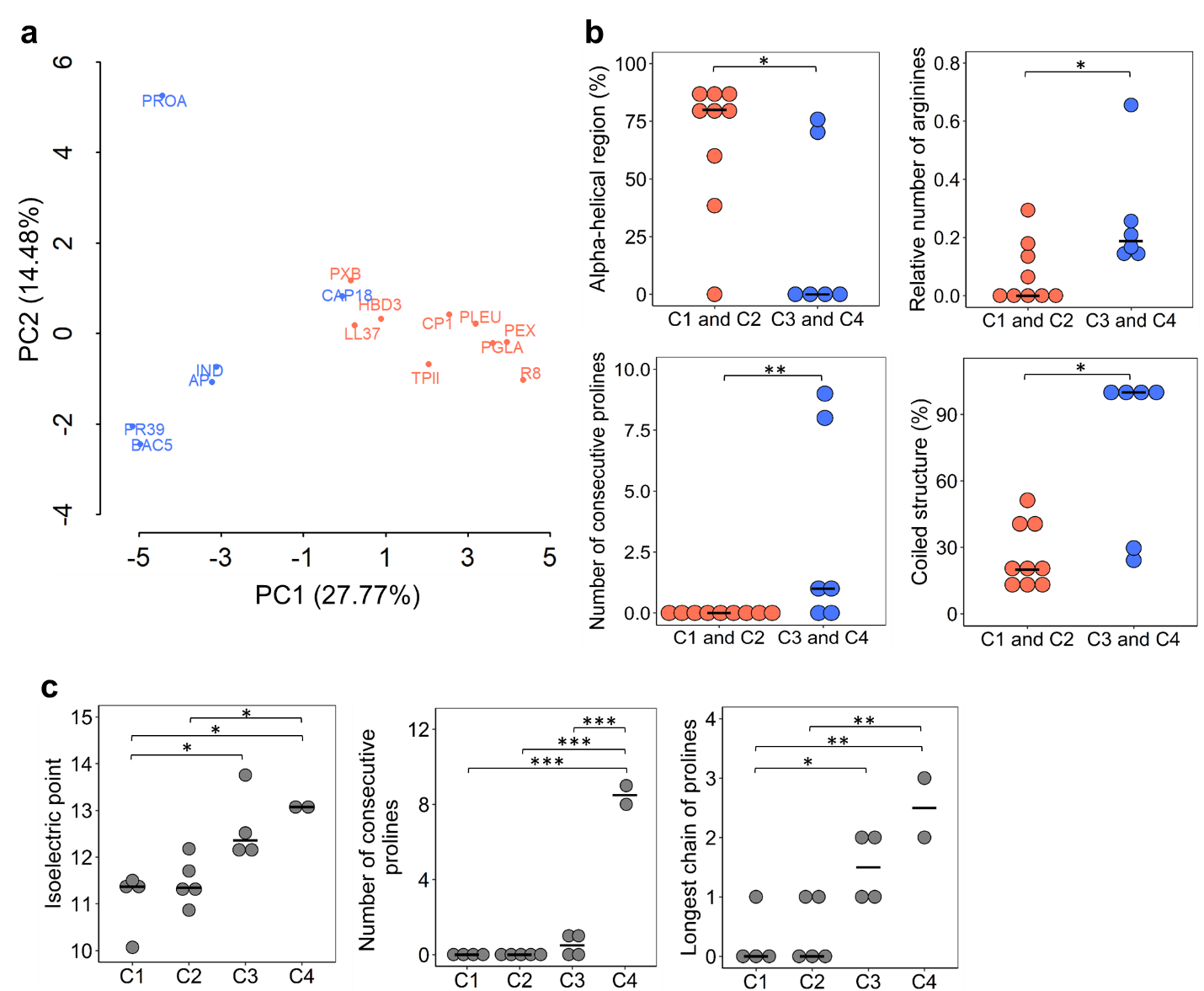


**Supplementary Figure 2.** **Physicochemical properties of AMPs that differentiate the clusters. a,** Principle component analysis (PCA) of the physicochemical properties differentiated AMPs in C1-C2 clusters (orange) from AMPs in C3-C4 clusters (blue) (*P* = 4.128*10^-5^, logistic regression, *n* = 15 AMPs). **b,** Four important physicochemical properties that differentiate membrane-targeting AMPs (orange, C1 and C2) from intracellular-targeting AMPs (blue, C3 and C4). AMPs from C1 and C2 clusters have more alpha-helical regions in their sequence compared to those from C3 and C4 clusters. AMPs from C3 and C4 clusters can be distinguished from those in clusters C1 and C2 based on their higher proline and arginine content. Significant differences from two-sided Mann–Whitney U test, * *P* = 0.0264, *P* = 0.0306 and *P* = 0.0147 for alpha-helical region (%), relative number of arginines and coiled structure (%), respectively. ** *P* = 0.008 for number of consecutive prolines (*n* = 9 for C1, C2 and n = 6 for C3, C4). **c,** Physicochemical properties that distinguish the clusters when AMPs in the four clusters are analysed separately (p<0.05 ANOVA, Tukey post hoc test). AMPs in clusters C1 and C2 show an especially low isoelectric point (significant differences: * *P* = 0.017, *P* = 0.014 and *P* = 0.04 for C1 vs C3, C1 vs C4 and C2 vs C4, respectively). AMPs from C4 cluster have an especially high number of consecutive prolines in their amino acid sequences compared to all other clusters (including cluster C3) (significant differences: *** *P* = 3.2*10^-11^, *P* = 9.8*10^-12^ and *P* = 1.1*10^-10^ for C1 vs C4, C2 vs C4 and C3 vs C4 respectively. AMPs from C1 and C2 can also be differentiated based on longest chain of prolines in their sequences (significant differences: * *P* = 0.039 for C1 vs C3. ** *P* = 0.0034 and *P* = 0.0044 for C1 vs C4 and C2 vs C4, respectively, *n* = 4, 5, 4 and 2 for AMPs from C1, C2, C3 and C4 clusters, respectively. Central horizontal lines represent median values. Source data are provided as Supplementary Data 3.


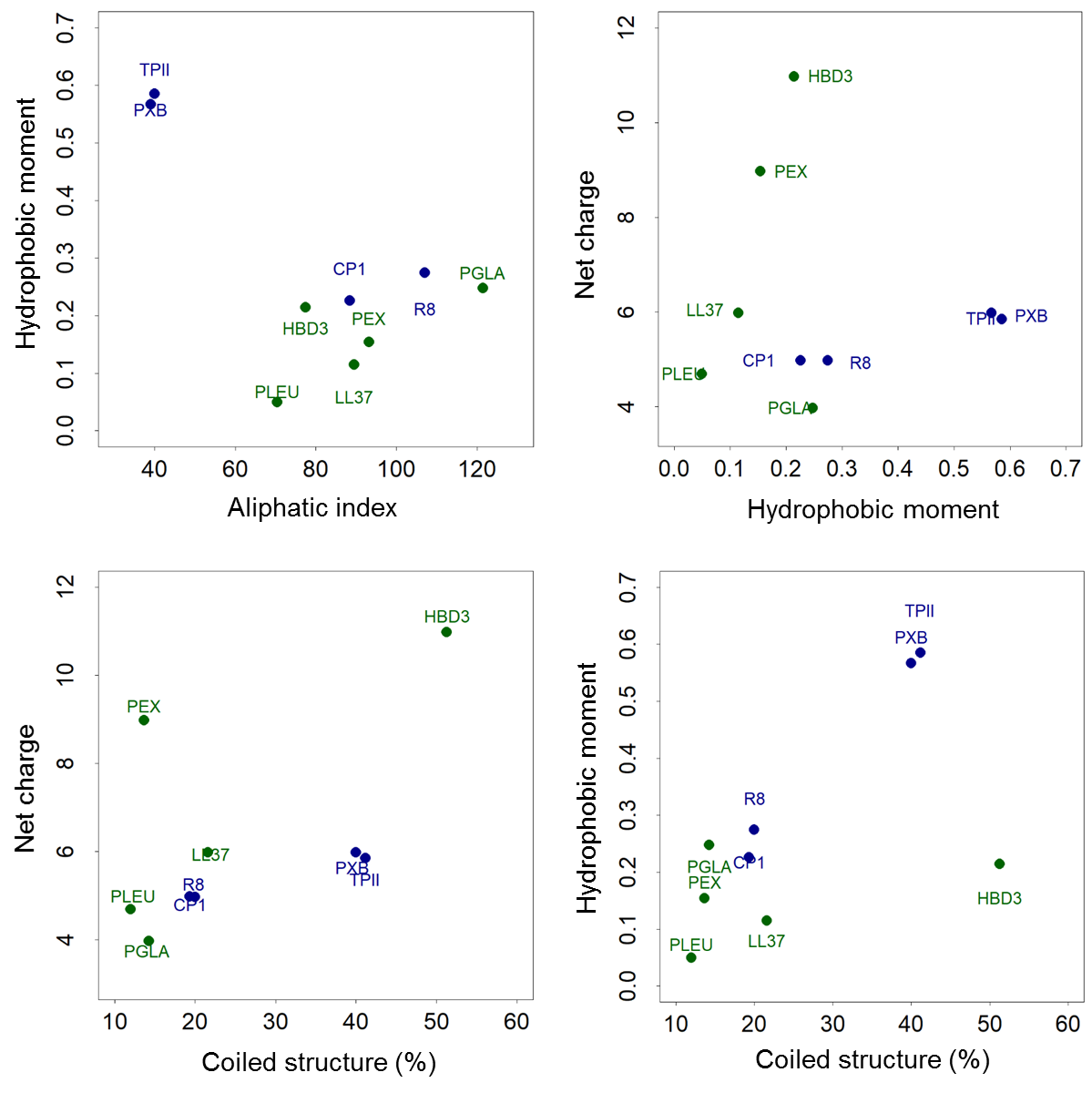


**Supplementary Figure 3. Physicochemical properties of AMPs that jointly separate C1 (blue) and C2 cluster peptides (green).** Although AMPs from both C1 and C2 clusters are known to target the bacterial cell membrane by creating pores in it, they group into different clusters on the basis of their chemical-genetic interactions (see main text and figures). Reassuringly, some physicochemical properties, when considered together, separate AMPs belonging to these two clusters (*P* = 0.0186 from two-sided logistic regression for all four combinations). *N* = 4 and 5 for AMPs from C1 and C2 clusters, respectively. Source data are provided as Supplementary Data 3.


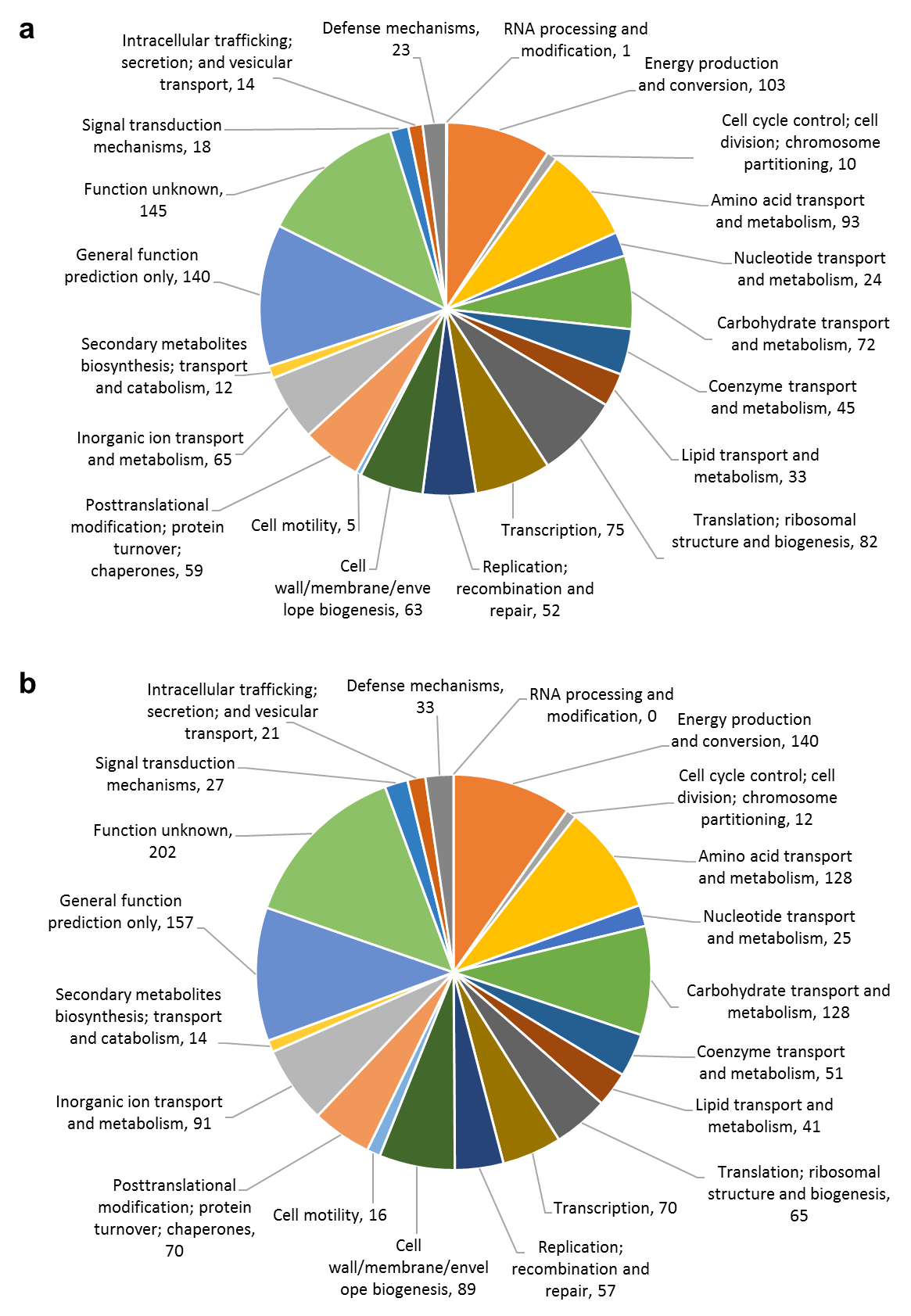


**Supplementary Figure 4. Distribution of Clusters of Orthologous Groups (COG) categories among the genes that (a) enhance resistance and (b) enhance sensitivity to at least one AMP in the chemical-genetic screen.** The value next to each COG category shows the total number of genes that belong to the corresponding COG category. Source data are provided as Supplementary Data 6.


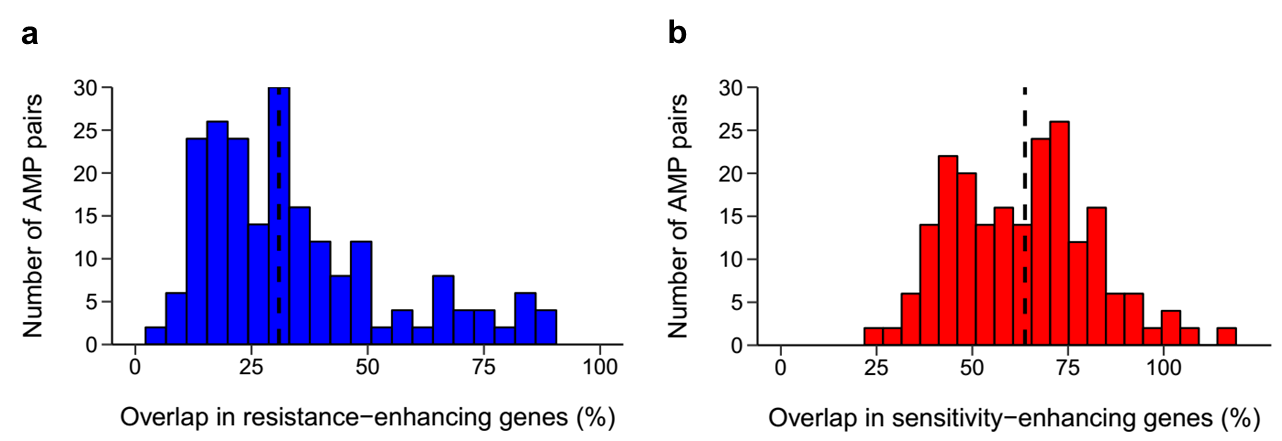


**Supplementary Figure 5. Resistance-enhancing genes overlap only to a limited extent between AMPs.** Distributions of overlaps in the **(a)** resistant (blue) and **(b)** sensitive (red) chemical-genetic interactions for all possible AMP pairs (*n* = 105 AMP pairs). Dashed line represents median value, 30.91 and 63.76 for resistance- and sensitivity-enhancing genes, respectively. Source data are provided as a Source Data file.


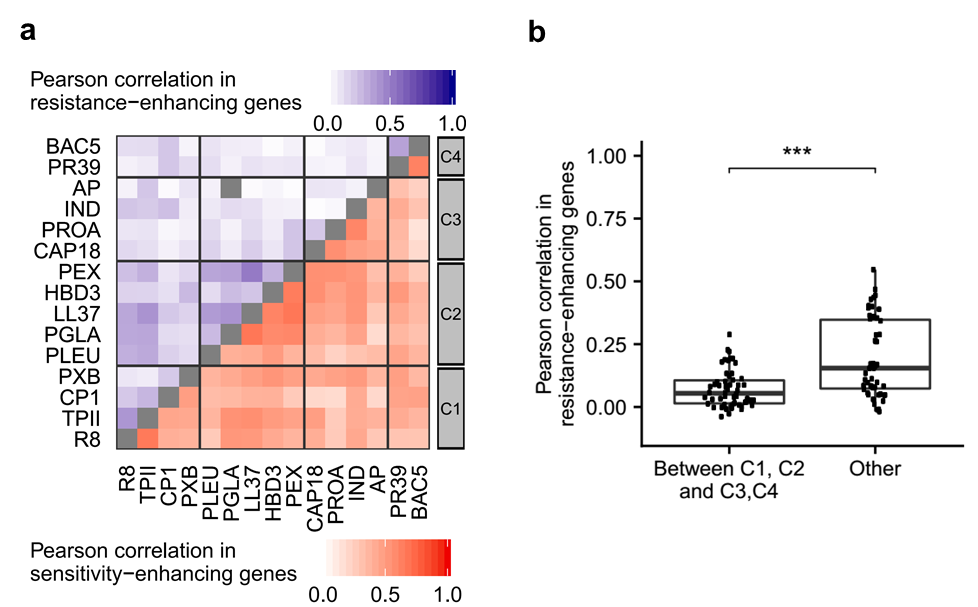


**Supplementary Figure 6. Large diversity of resistance determinants across AMPs as assessed by chemical-genetic interaction profile correlations.** **a,** Heatmap shows Pearson correlation coefficients of fold-change values calculated for resistance-enhancing genes (blue) and sensitivity-enhancing genes (red) between AMP pairs based on the overexpression screen (*n* = 210, that is, the number of AMP pairs). Pearson correlations for the resistant and sensitive parts of the chemical-genetic profiles were calculated by converting all fold-change values below our significance threshold to zero. For example, to measure the similarity of fold-change values for resistance interactions, we converted all fold changes <2 and P>0.05 in the profiles to zero and then calculated the correlation coefficient between these modified profiles. The darker the color the higher the correlation between the fold-change values between AMP pairs. Note that the correlations for resistance-enhancing genes are generally low, indicating largely dissimilar latent resistomes across AMPs. **b,** Similarity in the latent resistomes (genes enhancing resistance upon overexpression) between AMP pairs belonging to different chemical-genetic clusters. Similarity was calculated using Pearson correlation, as above. Significant difference: *** *P* = 2.3*10^-12^ from two-tailed Wilcoxon rank-sum test, *n* = 54 and *n* = 51 for between C1, C2 and C3, C4, and others, respectively. Boxplots show the median (center horizontal line), the first and third quartiles (bottom and top of box, respectively), with whiskers showing either the maximum (minimum) value or 1.5 times the interquartile range of the data. Source data are provided as a Source Data file.


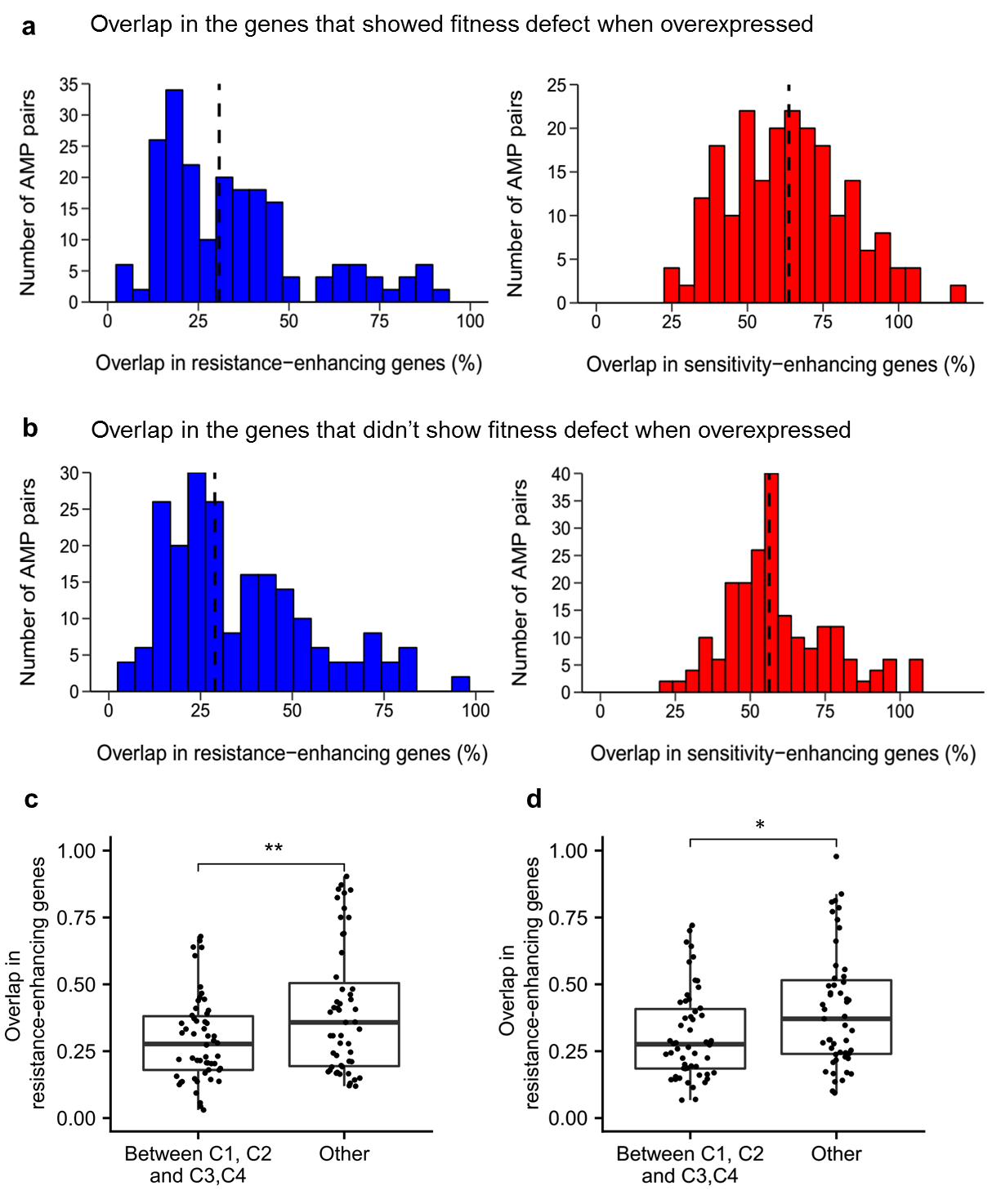


**Supplementary Figure 7. Distributions of overlaps in the chemical-genetic interactions for the resistant-enhancing (blue) and sensitivity-enhancing (red) genes for all possible AMP pairs (*n* = 105 AMP pairs) when the effect of the gene overexpression on growth rate was taken into consideration.** Histograms show distribution of overlaps in **a,** the genes that have been shown to cause fitness defect when overexpressed (median = 30.76 and 63.7 for resistance- and sensitivity-enhancing genes, respectively), and **b,** those genes that do not cause fitness defect (median = 28.88 and 56.32 for resistance- and sensitivity-enhancing genes, respectively). A previously published dataset was used to determine the effect of gene overexpression on cell growth^1^ (see Method). Dashed line represents median value. The overlap in the resistance-enhancing genes that **c,** cause fitness defect when overexpressed (significant difference: ** *P* = 0.0071 from two-tailed unpaired *t*-test, *n* = 54 and *n* = 51 for between C1, C2 and C3, C4, and others, respectively), and **d,** those that do not cause fitness defect genes between AMP pairs (significant difference: * *P* = 0.015 from two-tailed unpaired *t*-test, *n* = 54 and *n* = 51 for between C1, C2 and C3, C4, and others, respectively). Boxplots show the median (center horizontal line), the first and third quartiles (bottom and top of box, respectively), with whiskers showing either the maximum (minimum) value or 1.5 times the interquartile range of the data. Source data are provided as a Source Data file.


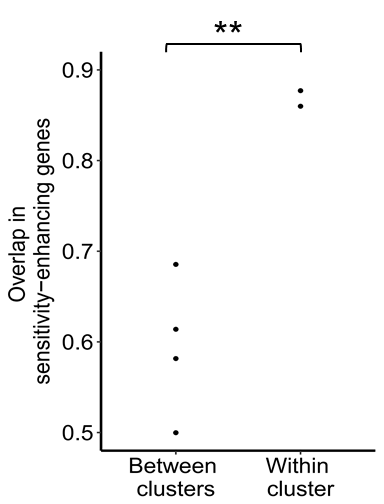


**Supplementary Figure 8. AMPs with similar mode of action show substantial overlap in their intrinsic resistomes**. On average, 87% of the 279 hypomorphic alleles overlapped between AMPs that have similar mode of action (i.e. within cluster) while only 59% of the 279 hypomorphic alleles were identical when functionally dissimilar AMPs were compared (i.e. between clusters). Significant difference: ** *P* = 0.0032 from two-tailed unpaired *t*-test, *n* = 2 and *n* = 4 for within and between clusters, respectively. Source data are provided as Supplementary Data 5.

**
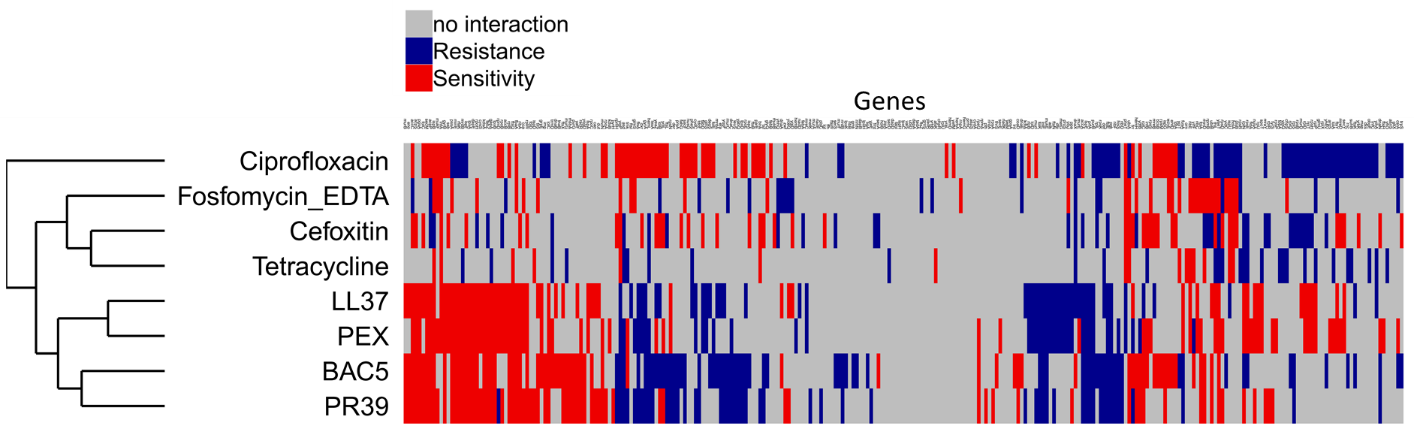
**

**Supplementary Figure 9. Antibiotics and AMPs have largely distinct chemical-genetic interaction profiles.** Heatmap and dendrogram (Ward’s method with Euclidean distances) show chemical-genetic interaction profiles across hypomorphic alleles of all essential *E. coli* genes in response to four antibiotics of different modes of action and four AMPs. The four tested AMPs cluster together indicating that the majority of the chemical-genetic interactions are specific to AMPs and not shared with antibiotics. Source data are provided as Supplementary Data 5.


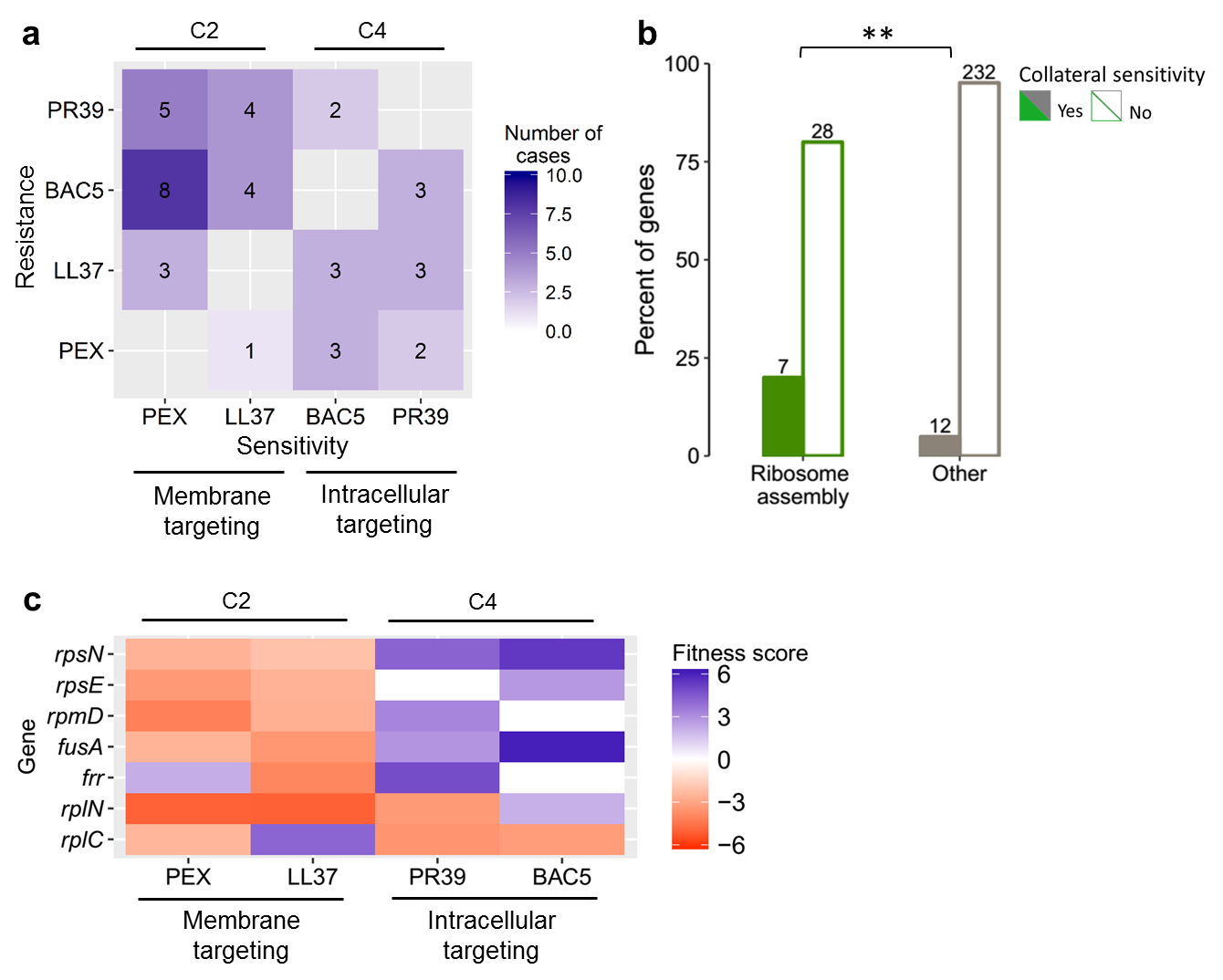


**Supplementary Figure 10. Hypomorphic alleles induce collateral sensitivity between intracellular-targeting and membrane-targeting AMPs.** **a,** Heatmap shows collateral sensitivity interaction between AMPs. Values in squares represent number of genes that show collateral sensitivity interactions. **b,** Hypomorphic alleles showing collateral sensitivity interactions were significantly enriched in functions related to ribosome assembly (significant difference: ** *P* = 0.004 from two-sided Fisher’s exact test, *n* = 35 and 244 for ribosome assembly related and other genes, respectively). **c,** Heatmap depicting fitness scores of the hypomorphic alleles enriched in ribosome assembly function. Blue and red colors represent resistance and sensitivity interactions, respectively. Source data are provided as Supplementary Data 5.


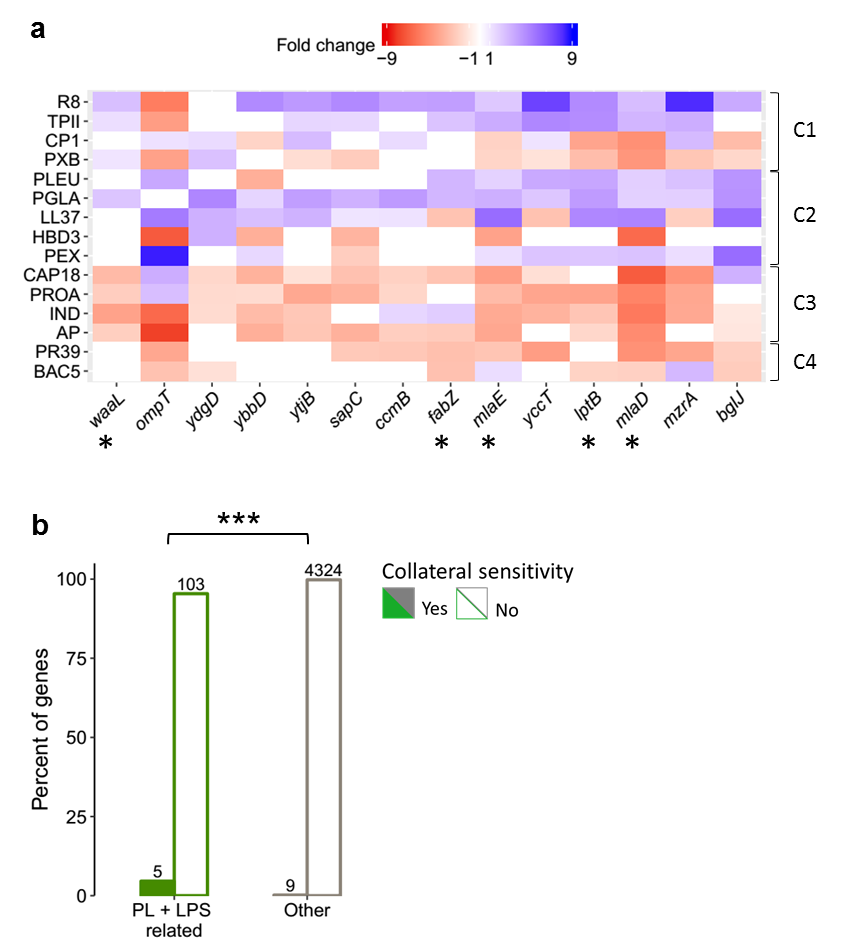


**Supplementary Figure 11. Phospholipid (PL) and lipopolysaccharide (LPS)-related genes frequently show collateral sensitivity interactions. a,** Heatmap presents the chemical-genetic profiles of genes enhancing resistance (blue) to at least 4 membrane-targeting AMPs and, simultaneously, sensitivity (red) to at least 4 intracellular-targeting AMPs, upon overexpression. Genes marked with asterisk have functions related to PL and LPS composition of the bacterial membranes. **b,** Overexpressed genes enhancing resistance to at least 4 membrane-targeting AMPs while at the same time sensitivity to at least 4 intracellular-targeting AMPs were significantly enriched in functions related to PL and LPS composition of the bacterial membranes (significant difference: *** *P* = 1.3*10^-5^ from two-sided Fisher’s exact test, *n* = 108 and 4,333 for PL+LPS related and other genes, respectively). Source data are provided as Supplementary Data 6.


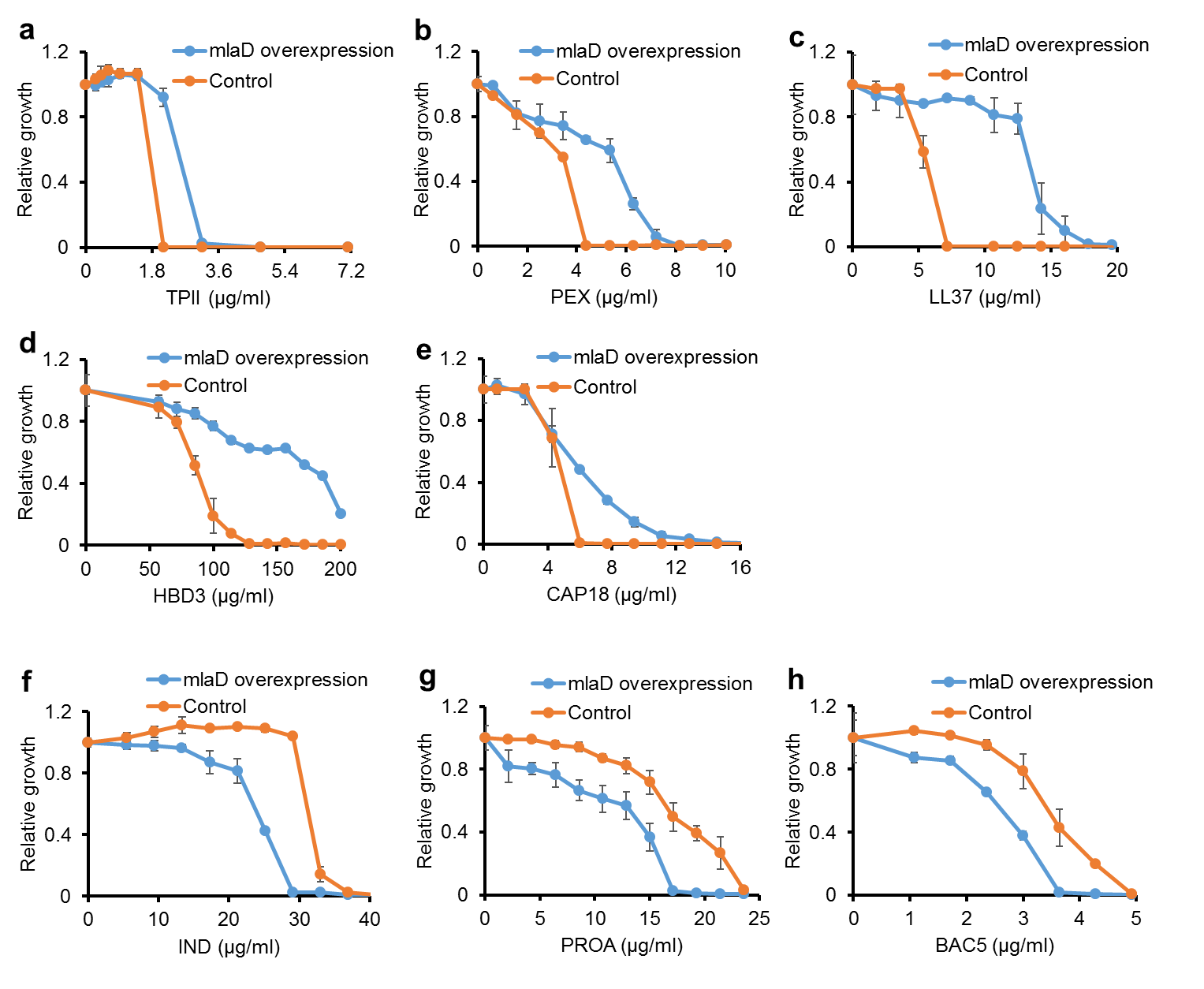


**Supplementary Figure 12. Effect of *mlaD* overexpression on bacterial susceptibility to membrane- and intracellular-targeting AMPs.** Overexpression of *mlaD* causes resistance to membrane-targeting **(a-e)** and sensitivity to intracellular-targeting AMPs **(f-h)**. Bacterial susceptibility to AMPs was tested by measuring MICs (see Methods). Growth is shown relative to growth in the absence of the given AMP (y-axis). The blue line represents the *mlaD* overexpression strain and the orange line represents the control strain (wild-type *E. coli* BW25113 containing the empty pCA24N vector). Error bars indicate standard errors based on three biological replicates. Source data are provided in the Source Data file.


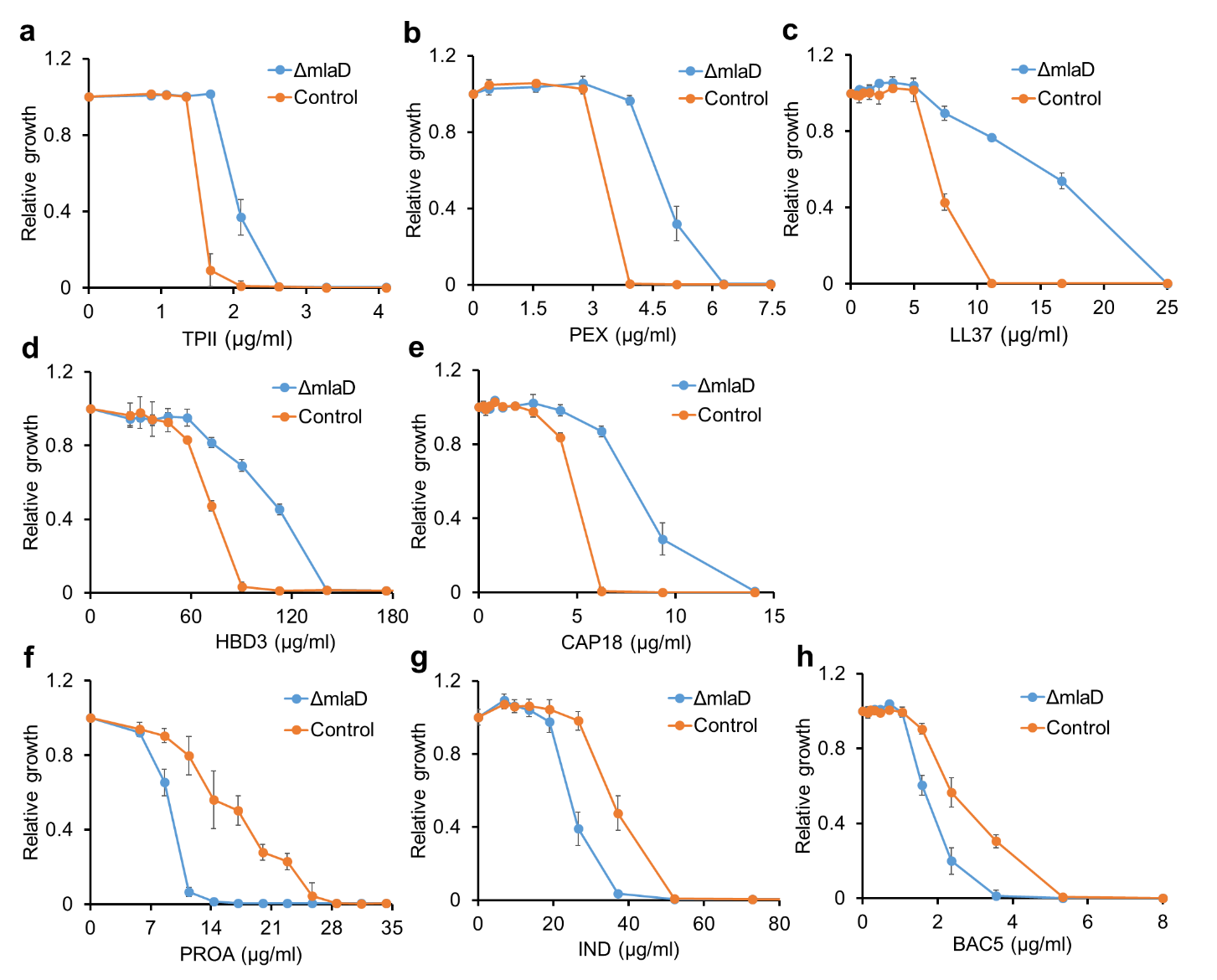


**Supplementary Figure 13. Antimicrobial susceptibility of *mlaD* knockout mutant to membrane- and intracellular-targeting AMPs.** *mlaD* knockout mutant shows resistance to membrane-targeting **(a-e)** and sensitivity to intracellular-targeting AMPs **(f-h)**. The bacterial susceptibility of the mutant to AMPs was tested by MIC determination. Growth is shown relative to growth in the absence of the corresponding AMP. The blue line represents the *mlaD* knockout strain and the orange line represents the control strain (wild-type *E. coli* BW25113). Error bars indicate the standard errors based on three biological replicates. Source data are provided in the Source Data file.


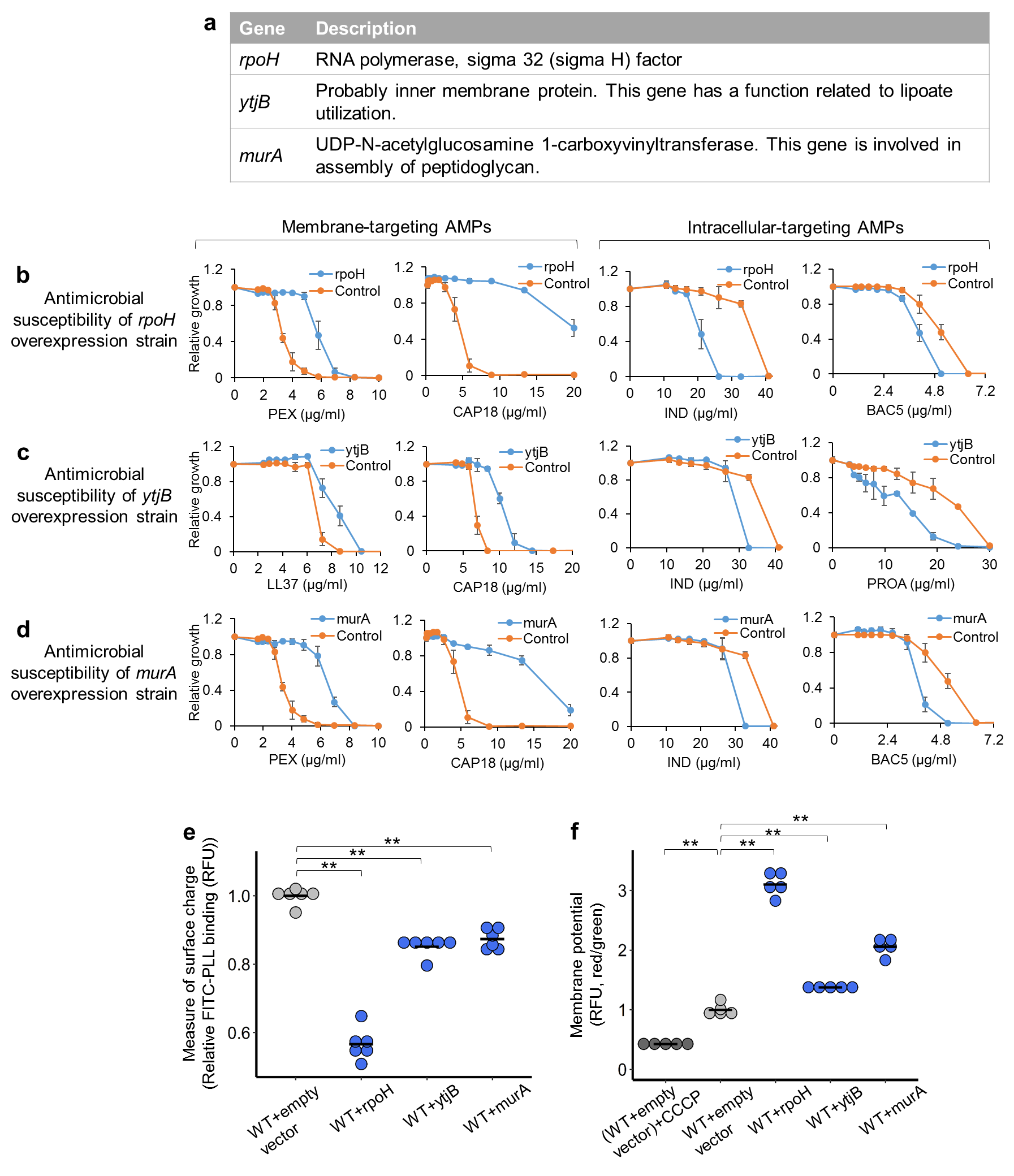


**Supplementary Figure 14.** **Antimicrobial susceptibility of *rpoH*, *ytjB* and *murA* overexpression strains to membrane- and intracellular-targeting AMPs. (b,c,d)** Strains overexpressing *rpoH*, *ytjB* and *murA* genes all showed resistance to membrane-targeting and sensitivity to intracellular-targeting AMPs. Bacterial susceptibility to AMPs was tested by MIC measurements. Growth is shown relative to growth in the absence of drug. Wild-type *E. coli* BW25113 containing the empty pCA24N vector was used as a control strain. Error bars indicate standard errors based on three biological replicates. **e,** Decreased net negative surface charge of the *rpoH*, *ytjB* and *murA* overexpression strains. Significant differences: ** *P* = 0.0021, *P* = 0.0021 and *P* = 0.0021 for WT+empty vector vs WT+*rpoH* overexpression, WT+empty vector vs WT+*ytjB* overexpression, WT+empty vector vs WT+*murA* overexpression strains, respectively, from two-sided Mann–Whitney U tests, *n* = 6 biological replicates for each genotype. Charge measurement was done using FITC-labelled poly-L-lysine (FITC-PLL) assay where the fluorescence signal is proportional to the binding of the FITC-PLL molecules. Lower binding of FITC-PLL indicates less net negative surface charge of the outer bacterial membrane (see Methods). **f,** Increased membrane potential of *rpoH*, *ytjB* and *murA* overexpression strains. Significant differences: ** *P* = 0.0079, *P* = 0.0079, *P* = 0.0079 and *P* = 0.0079 for WT+empty vector CCCP control vs WT+empty vector, WT+empty vector vs WT+*rpoH* overexpression, WT+empty vector vs WT+*ytjB* overexpression, and WT+empty vector vs WT+*murA* overexpression strains, respectively, from two-sided Mann–Whitney U test, *n* = 5 biological replicates for each genotype. Relative membrane potential was measured by determining relative fluorescence (RFU) using a carbocyanine dye DiOC2(3) assay (see Methods). Red/green ratios were calculated using population mean fluorescence intensities. Wild-type *E. coli* BW25113 carrying the empty vector treated with CCCP was used as an experimental control for diminished membrane potential. Central horizontal lines represent mean values. Raw data are in Supplementary Figure 16 and in Source Data file.


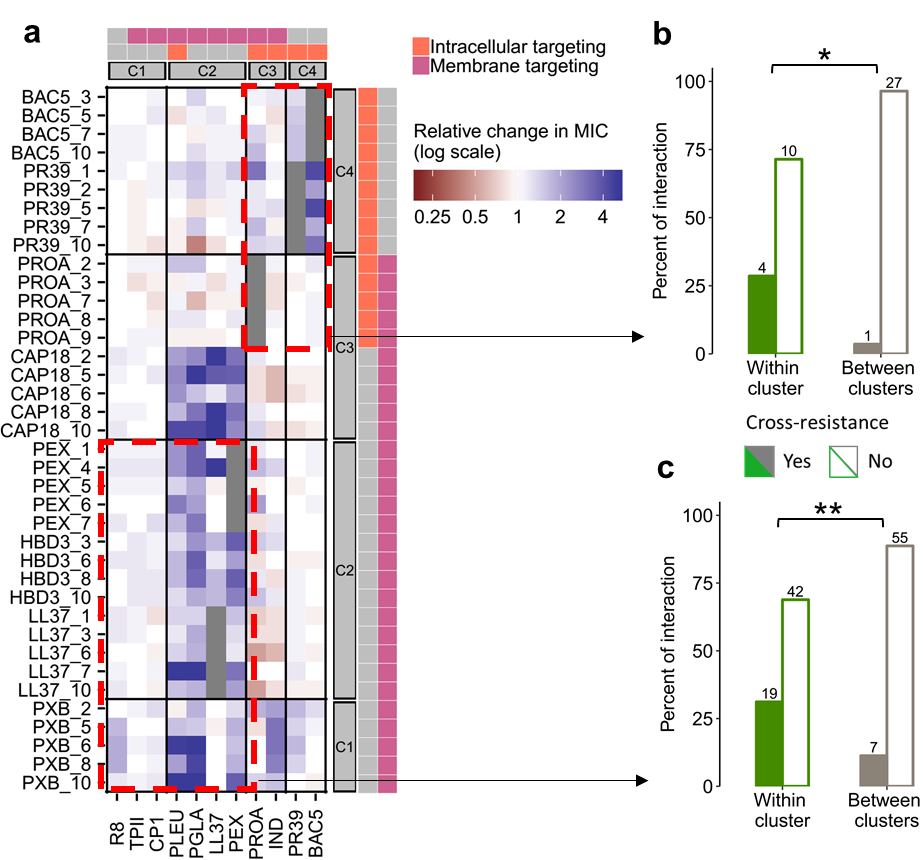


**Supplementary Figure 15. Chemical-genetic clustering provides insights into cross-resistance patterns between AMPs.** **a**, Heatmap shows cross-resistance spectra of AMP-evolved lines (rows) towards a set of 11 AMPs (columns). Relative change in MIC was determined for each evolved line by comparing its MIC to the ancestral cell line (control). Heatmap depicts relative MIC changes (log2-scaled values) with blue, red and grey colors indicating cross-resistance, collateral sensitivity and not applicable (i.e. resistance to cognate AMPs are not shown), respectively. **b**, Cross-resistance interactions are enriched between intracellular-targeting AMPs that are in the same chemical-genetic clusters (i.e. AMP pairs within C3 and within C4 clusters) (Significant difference: * *P* = 0.0353 from two-sided Fisher’s exact test, *n* = 14 and 28 for within cluster and between clusters, respectively). CAP18-adaped lines were not included in the analysis because CAP18 is known as a membrane-targeting AMP in the literature. **c**, Cross-resistance interactions are enriched between membrane-targeting AMPs that are in the same chemical-genetic clusters (i.e. AMP pairs within C1 and within C2 clusters) (Significant difference: ** *P* = 0.0082 from two-sided Fisher’s exact test, *n* = 61 and 62 for within cluster and between clusters, respectively). Overall, these results demonstrate that chemical-genetic clustering provides additional insights into the cross-resistance patterns beyond what can be gained by the broad mode of action of AMPs (i.e. intracellular- versus membrane-targeting). Source data are provided as a Source Data file.


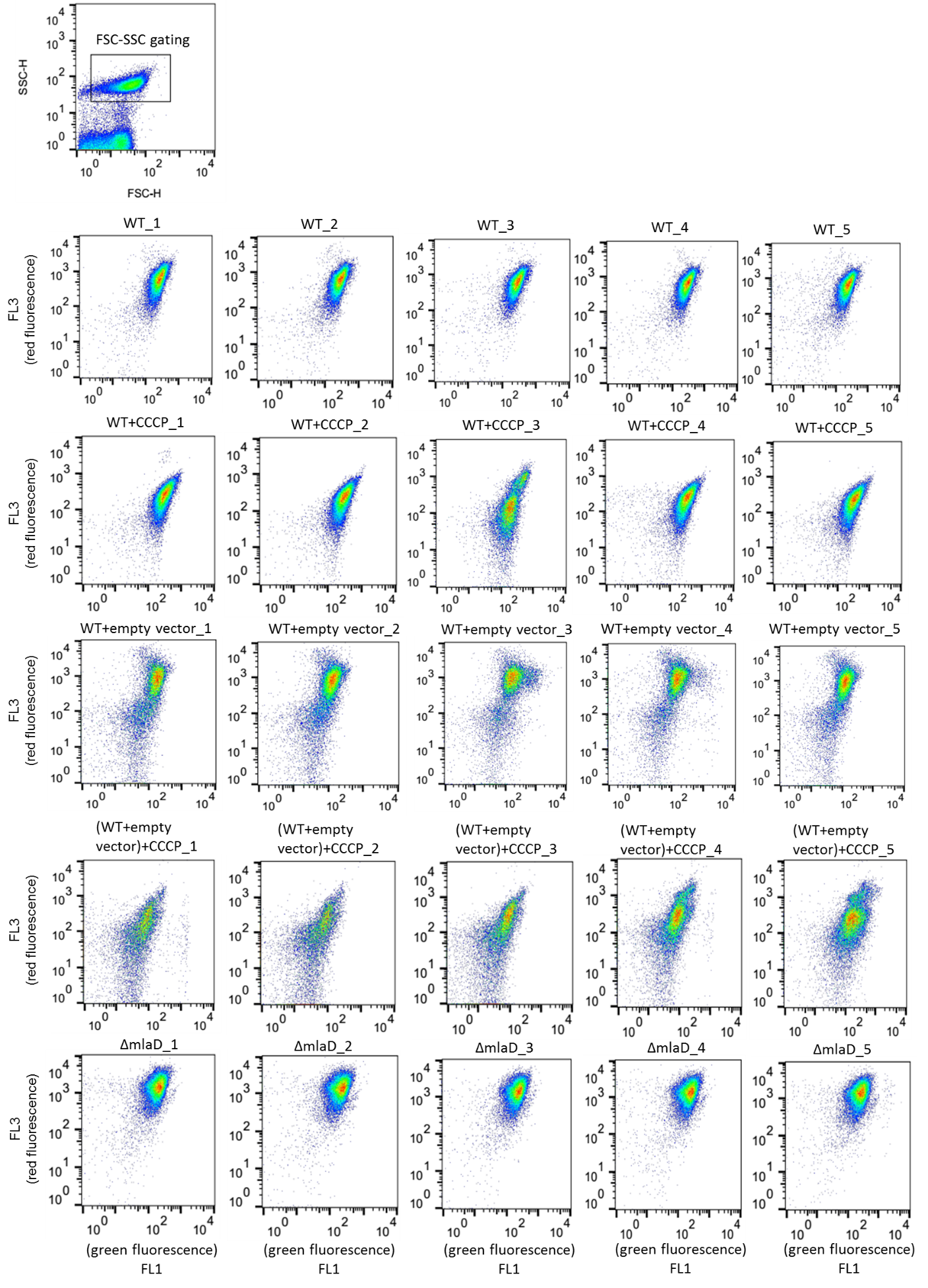


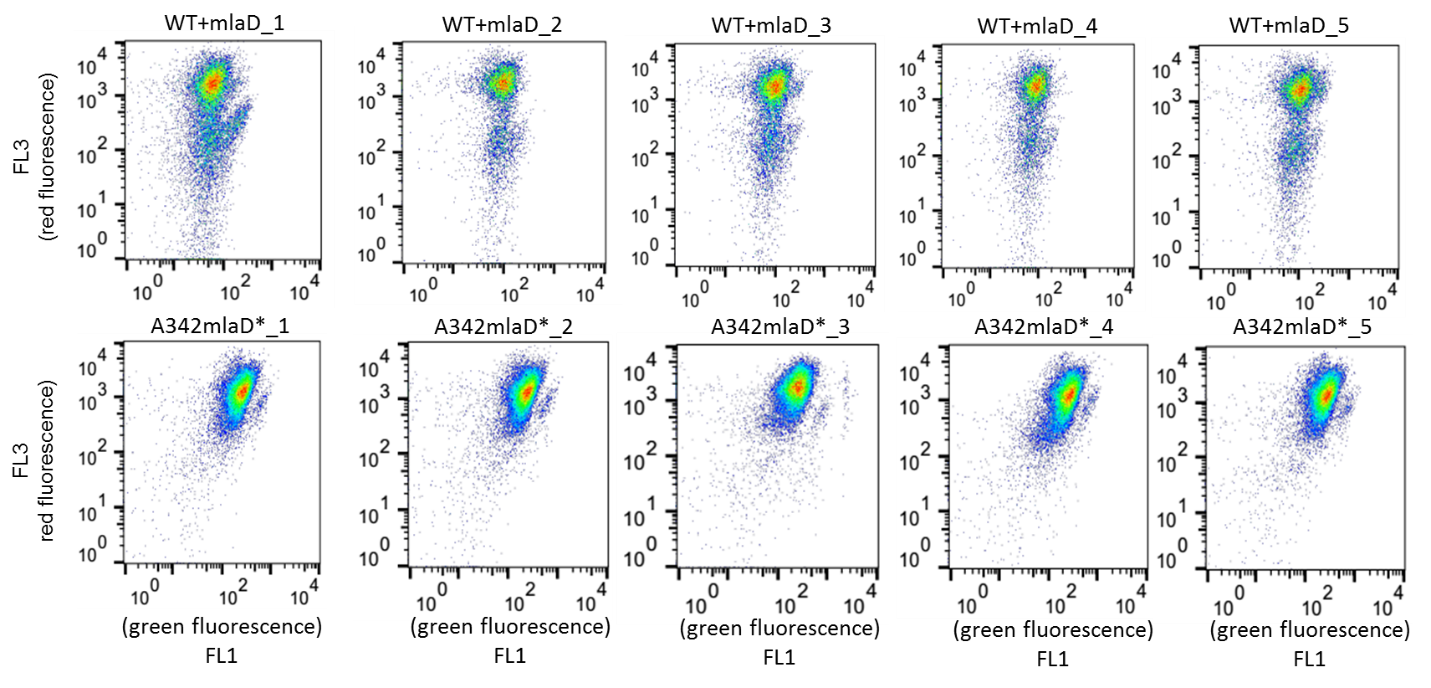


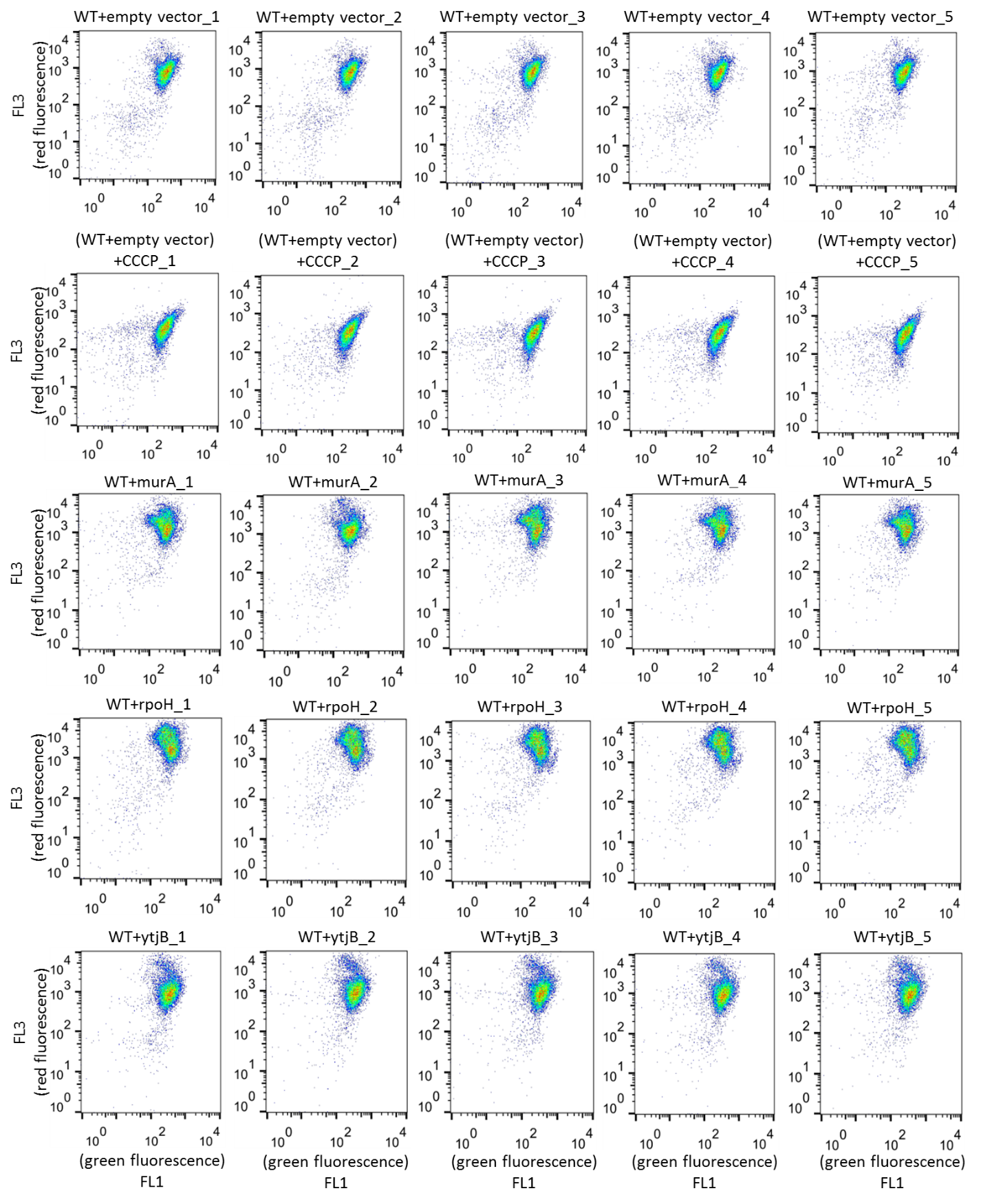


**Supplementary Figure 16**. **Membrane potential measurement.** Red (FL3-H) vs green (FL1-H) fluorescence scatter plots showing cells treated with the DiOC_2_(3) dye (membrane potential indicator, see Methods). For each strain we had five biological replicates. Top figure showed gating strategy where cells were gated using forward and side scatter properties. Raw data are provided as Supplementary Data 8.


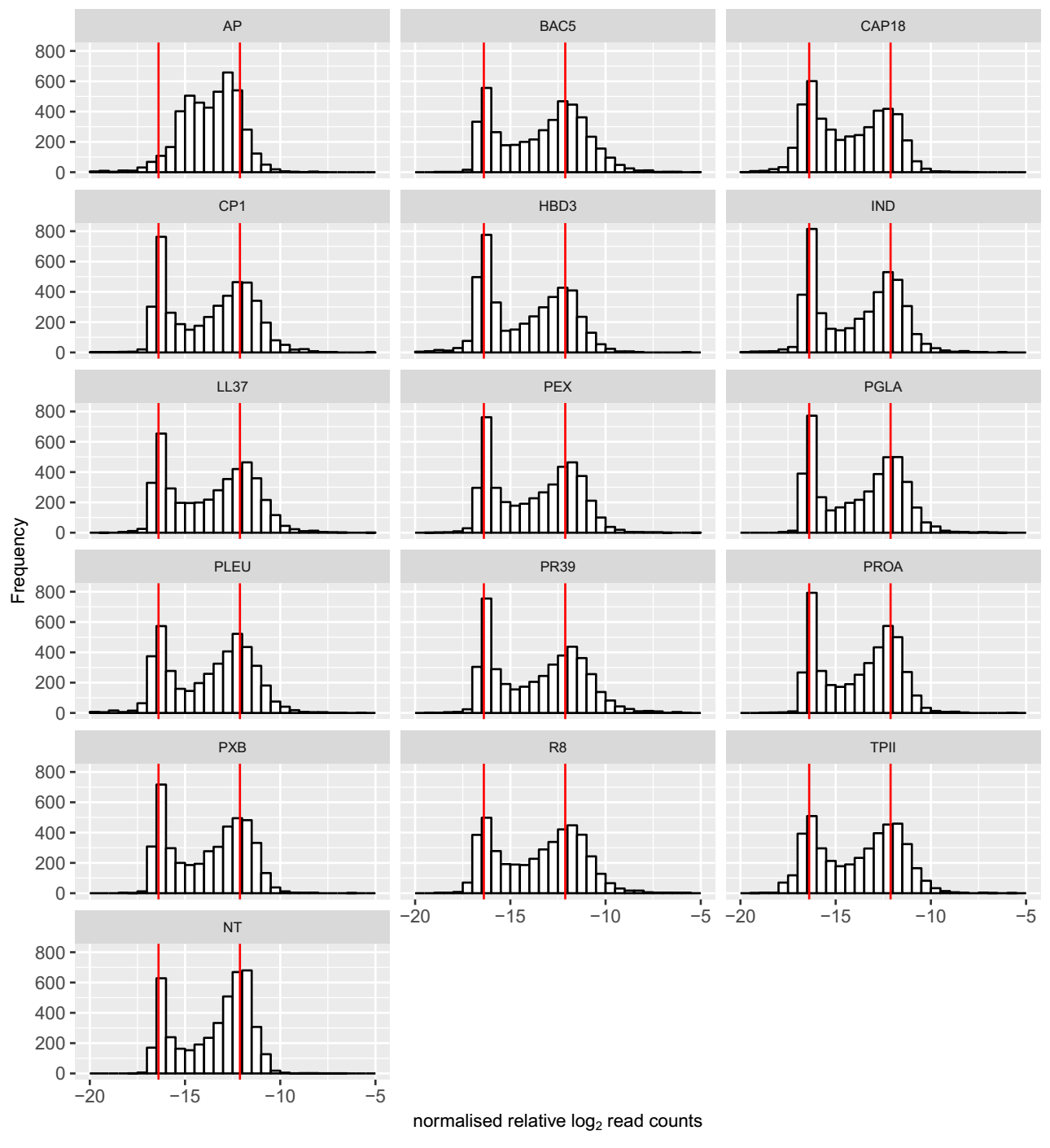


**Supplementary Figure 17**. **Distribution of the normalized read counts of plasmids overexpressing *E. coli* ORFs in the absence (NT) and presence of AMPs.** Following data processing (see Methods), the transformed relative read counts showed bimodal distributions (peaks of the two modes are marked with red lines). The lower mode of the distribution corresponds to ORFs that were not present in the sample. The upper mode represents those ORFs whose growth was unaffected by overexpression (i.e. no fitness effect).

**Supplementary Tables**

**Supplementary Table 1. Collected examples of *E. coli* gene overexpression from the literature as a confirmation of our chemical-genetic screen results.**

| **Gene** | **AMP** | **Overexpression phenotype** | |
| --- | --- | --- | --- |
|  |  | **Literature** | **Chemical-genetic profile** |
| *ptrB* | BAC5 | Resistance^2^ | Confirmed |
|  | PR39 | Resistance^2^ | Confirmed |
| *marA* | PXB | Resistance^3^ | Confirmed |
|  | LL37 | Resistance^3^ | X |
| *ompT* | PROA | Resistance^4^ | Confirmed |
|  | LL37 | Resistance^4^ | Confirmed |
| *arnT* | PXB | Resistance^5^ | X |
| *nlpE* | PROA | Resistance^6^ | Confirmed |
| *eptA* | PXB | Resistance^7^ | Confirmed |
| *pmrD* | PXB | Resistance^8^ | X |
| *sbmA* | PR39 | Sensitivity^9^ | Confirmed |
| *waaY* | CAP18 | Sensitivity^10^ | Confirmed |
| *prfA* | AP | Resistance^11^ | X |

**Supplementary Data files**

**Supplementary Data 1. A comprehensive catalogue of previously reported genes (based on literature mining) that modulate bacterial susceptibility to antimicrobial peptides (AMPs).**

Provided in a separate Excel spreadsheet.

**Supplementary Data 2. Complete dataset of chemical-genetic interactions of ~4400 mutants across 15 different antimicrobial peptides (AMPs).**

Provided in a separate Excel spreadsheet.

**Supplementary Data 3. List of the physicochemical properties of the antimicrobial peptides (AMPs) used in this study.**

Provided in a separate Excel spreadsheet.

**Supplementary Data 4. Gene ontology (GO) enrichment analysis of the genes enhancing resistance and sensitivity to antimicrobial peptides (AMPs).**

Provided in a separate Excel spreadsheet.

**Supplementary Data 5. Complete dataset of chemical-genetic interactions of hypomorphic alleles in response to antimicrobial peptides (AMPs) and antibiotics.**

Provided in a separate Excel spreadsheet.

**Supplementary Data 6. List of resistance- and sensitivity-enhancing genes and their collateral sensitivity (CS) interactions.**

Provided in a separate Excel spreadsheet.

**Supplementary Data 7. Detailed information (based on literature mining) of the antimicrobial peptides (AMPs) used in this study.**

Provided in a separate Excel spreadsheet.

**Supplementary Data 8. Raw dataset of red (FL3-H) and green (FL1-H) fluorescence from membrane potential measurement.**

Provided in a separate Excel spreadsheet.

# References for Supplementary information

1. Chen, H. *et al.* Genome-Wide Quantification of the Effect of Gene Overexpression on Escherichia coli Growth. *Genes (Basel).* **9**, 414 (2018).

2. Mattiuzzo, M. *et al.* Proteolytic activity of Escherichia coli oligopeptidase B against proline-rich antimicrobial peptides. *J. Microbiol. Biotechnol.* (2014). doi:10.4014/jmb.1310.10015

3. Warner, D. M. & Levy, S. B. Different effects of transcriptional regulators MarA, SoxS and Rob on susceptibility of Escherichia coli to cationic antimicrobial peptides (CAMPs): Rob-dependent CAMP induction of the marRAB operon. *Microbiology* **156**, 570–578 (2010).

4. Stumpe, S., Schmid, R., Stephens, D. L., Georgiou, G. & Bakker, E. P. Identification of OmpT as the protease that hydrolyzes the antimicrobial peptide protamine before it enters growing cells of Escherichia coli. *J. Bacteriol.* (1998).

5. Trent, M. S. *et al.* Accumulation of a Polyisoprene-linked Amino Sugar in Polymyxin-resistant Salmonella typhimurium and Escherichia coli. *J. Biol. Chem.* **276**, 43132–43144 (2001).

6. Griffin NW. Identification of Novel Genetic Mechanisms Required for Bacterial Resistance to Antimicrobial Peptides. (<https://repository.asu.edu/attachments/110499/content/Griffin_asu_0010E_12909.pdf>) (2013).

7. Olaitan, A. O., Morand, S. & Rolain, J. M. Mechanisms of polymyxin resistance: Acquired and intrinsic resistance in bacteria. *Frontiers in Microbiology* (2014). doi:10.3389/fmicb.2014.00643

8. Roland, K. L., Esther, C. R. & Spitznagel, J. K. Isolation and characterization of a gene, pmrD, from Salmonella typhimurium that confers resistance to polymyxin when expressed in multiple copies. *J. Bacteriol.* **176**, 3589–3597 (1994).

9. Pranting, M., Negrea, A., Rhen, M. & Andersson, D. I. Mechanism and Fitness Costs of PR-39 Resistance in Salmonella enterica Serovar Typhimurium LT2. *Antimicrob. Agents Chemother.* **52**, 2734–2741 (2008).

10. Lázár, V. *et al.* Antibiotic-resistant bacteria show widespread collateral sensitivity to antimicrobial peptides. *Nat. Microbiol.* **3**, 718–731 (2018).

11. Matsumoto, K. *et al.* In vivo target exploration of apidaecin based on Acquired Resistance induced by Gene Overexpression (ARGO assay). *Sci. Rep.* **7**, 12136 (2017).
