# Supplementary material for: Chemical-genetic profiling reveals limited cross-resistance between antimicrobial peptides with different modes of action
Source: Nat Commun. 2019 Dec 16;10:5731. doi: 10.1038/s41467-019-13618-z (PMC6915728; doi:10.1038/s41467-019-13618-z)
Supplement: Supplementary file 3 — Description of Additional Supplementary Files [file 41467_2019_13618_MOESM3_ESM.docx]

**Description of Additional Supplementary Files**

File Name: **Supplementary Data 1**

Description: **A comprehensive catalogue of previously reported genes (based on literature mining) that modulate bacterial susceptibility to antimicrobial peptides (AMPs).**

File Name: **Supplementary Data 2**

Description: **Complete dataset of chemical-genetic interactions of ~4400 mutants across 15 different antimicrobial peptides (AMPs).**

File Name: **Supplementary Data 3**

Description: **List of the physicochemical properties of the antimicrobial peptides (AMPs) used in this study.**

File Name: **Supplementary Data 4**

Description: **Gene ontology (GO) enrichment analysis of the genes enhancing resistance and sensitivity to antimicrobial peptides (AMPs).**

File Name: **Supplementary Data 5**

Description: **Complete dataset of chemical-genetic interactions of hypomorphic alleles in response to antimicrobial peptides (AMPs) and antibiotics.**

File Name: **Supplementary Data 6**

Description: **List of resistance- and sensitivity-enhancing genes and their collateral sensitivity (CS) interactions.**

File Name: **Supplementary Data 7**

Description: **Detailed information (based on literature mining) of the antimicrobial peptides (AMPs) used in this study.**

File Name: **Supplementary Data 8**

Description: **Raw dataset of red (FL3-H) and green (FL1-H) fluorescence from membrane potential measurement.**
